# Association between suspected Zika virus disease during pregnancy and giving birth to a newborn with congenital microcephaly: a matched case–control study

**DOI:** 10.1186/s13104-017-2796-1

**Published:** 2017-09-06

**Authors:** Ticiane Henriques Santa Rita, Renata Barcelos Barra, Gisele Pasquali Peixoto, Pedro Goes Mesquita, Gustavo Barcelos Barra

**Affiliations:** 1 Sabin Laboratory, SAAN Quadra 03, Lotes 165 e 245, Federal District, Brasília, Brazil; 2 University of Brasília, Federal District, Brasilia, Brazil; 3 Secretaria do Estado da Saúde, Federal District, Brasília, Brazil

**Keywords:** Zika virus, Microcephaly, Case–control study

## Abstract

**Objective:**

In early 2015, an outbreak of an acute exanthematous illness with dengue-like symptoms occurred in northeastern Brazil. By the end of the same year, an unexpected increase in the number of cases of microcephaly was observed in the region. The microcephaly outbreak cause was unknown and rumors pointing to various potential causes arose. Since we were unaware at the time if this scenario would attract the interest of the broader scientific community, due to the neglected regions associated and as often happens with many others health conditions related to infectious diseases in Latin America. This coupled with the fact that diagnostic testing for Zika virus was not available, prompted us to design a study that could demonstrate the correlation between the development of an exanthematous illness with Zika-like symptoms during pregnancy and the delivery of a newborn with congenital microcephaly.

**Results:**

Mothers who experienced symptoms associated with the Zika virus during pregnancy had 10 times higher odds of delivering newborns with congenital microcephaly when compared with mothers who did not exhibit Zika-like symptoms. Thus, the acute exanthematous illness outbreak could be associated with the congenital microcephaly outbreak. We could not distinguish which virus caused the acute exanthematous illness in the study subjects (Zika, dengue or chikungunya), but these results could help to reduce the misquided speculation in regards to the cause of the microcephaly and could have expedited public health policies intended for controlling the mosquito vector. In addition to the lower head circumference, microcephalic neonates also had lower thoracic circumference, lower height and lower weight compared to non-microcephalic babies suggesting intrauterine growth restriction. Additionally, we found borderline association between mothers classified as homemakers and, who had past dengue infections with microcephaly. Prior contraction of dengue virus seems to play a role in the risk for the condition reflecting the domestication of the *Aedes Aegypti* and the enhancement of the Zika virus infection by dengue antibodies, respectively. The limitations of this study are: (a) participants recall bias, (b) absence of laboratory test results for Zika virus and other arboviruses and (c) incomplete test results for other pathogens that could lead to microcephaly.

The study protocol was registered at ClinicalTrial.gov under the identifier NCT02741882. Registered on April 13th, 2016

**Electronic supplementary material:**

The online version of this article (doi:10.1186/s13104-017-2796-1) contains supplementary material, which is available to authorized users.

## Background

In early 2015, an outbreak of an acute exanthematous illness with dengue-like symptoms occurred in northeastern Brazil [1, 2]. The condition was characterized, mainly, by rash, headache, joint pain, conjunctivitis and other symptoms that included mild fever and fatigue [1, 2]. Further investigation revealed the cocirculation of Zika, dengue and chikungunya viruses in the region and each one can be responsible for causing diseases of this clinical type [1–3].

In late 2015, an unexpected increase in the number of microcephaly notifications was observed in the northeastern Brazil [4]. The cause was unknown and rumors citing many potential causes arose including the Zika virus, along with genetically modified mosquitoes, larvicide in drinking water, rubella vaccine, pertussis vaccine and underreporting of microcephaly cases for years [5]. However, the consensus suspect was the Zika virus, because its mRNA was found in the amniotic fluid samples of two pregnant women whose fetuses were diagnosed with microcephaly [6].

As with other past outbreaks in Latin American, we did not know if the microcephaly increase would attract the interest of local and international scientific communities. Moreover, at the time, a diagnostic test that could be used to identify past exposure to Zika virus was not available [7]. Thus, we developed a case–control study to investigate the link between having an acute exanthematous illness accompanied with others Zika-like symptoms during gestation, and subsequently giving birth to a newborn with congenital microcephaly. The main limitation of the proposed research model, based on clinical signs and symptoms, is the absence of a diagnostic tests for Zika virus and/or other arboviruses.

Fortunately, the World Health Organization declared the microcephaly outbreak a Public Health Emergency of International concern in 2016 [8], thereby drawing attention to the association between Zika virus and congenital malformations. This major action has lead to an abundance of research and, today, it is well established that Zika virus infections during pregnancy are responsible for causing the Zika congenital syndrome, which includes microcephaly and other malformations [9–11].

As a result of all this, the impact of this study was minimized slightly due to additional, available significant data that utilized definitive diagnostic testing for Zika. However, we did find an association between the studied exposure (suspected Zika virus disease) and the outcome (congenital microcephaly). These findings provide valuable insights that could be useful in assisting health care providers to estimate the risk of microcephaly by assessing clinical signs and symptoms experienced by the mother during the pregnancy which, until now, remains a major challenge. Identification of Zika virus can be difficult due to the virus’s ability to cross-react with dengue virus and other flaviviruses tests and/or when sufficient diagnostic tests for Zika are not available as is often the case in poor underserved regions.

## Main text

We carried out a retrospective 1:2 matched case–control study among parturients admitted at the public maternity hospital, “Nossa Senhora de Lourdes”, located in Aracaju, in the state of Sergipe, on the northeast coast of Brazil. The aim of the study was to identify a correlation between the development of an acute exanthematous illness displaying Zika-like symptoms and pregnancies which resulted in the delivery of a new baby born with congenital microcephaly.

From September 1st, 2015 to January 5th, 2016, the maternity hospital reported to Brazil’s ministry of healthy that 64 newborn babies were delivered with probable congenital microcephaly. The mothers of those babies were eligible to be analyzed as cases. Mothers from the same maternity whom delivered newborns without the condition were eligible to be a part of the control group.

Maternal/neonate data was obtained from the medical records. The exclusion criteria were: neonates with head circumference in the normal range for the gestational age according to WHO guidelines [12], mother with prenatal detection of syphilis, human immunodeficiency virus, toxoplasmosis, cytomegalovirus or rubella (if available), neonate with diagnoses of other genetic syndromes and/or lack of data.

Of the 64 neonates born with probable congenital microcephaly, 21 had head circumferences in the normal range and one was diagnosis with Seckel syndrome. Two mothers had tested positive for syphilis (VDRL) and one for toxoplasmosis (IgM) during pregnancy. Three neonates/mothers had medical records that were substantially incomplete. All of them were excluded. For the included subjects, data for syphilis and human immunodeficiency virus were complete. However, data for toxoplasmosis, cytomegalovirus and rubella were partially complete (Additional file 1: Table S1).

**Fig. 1:**
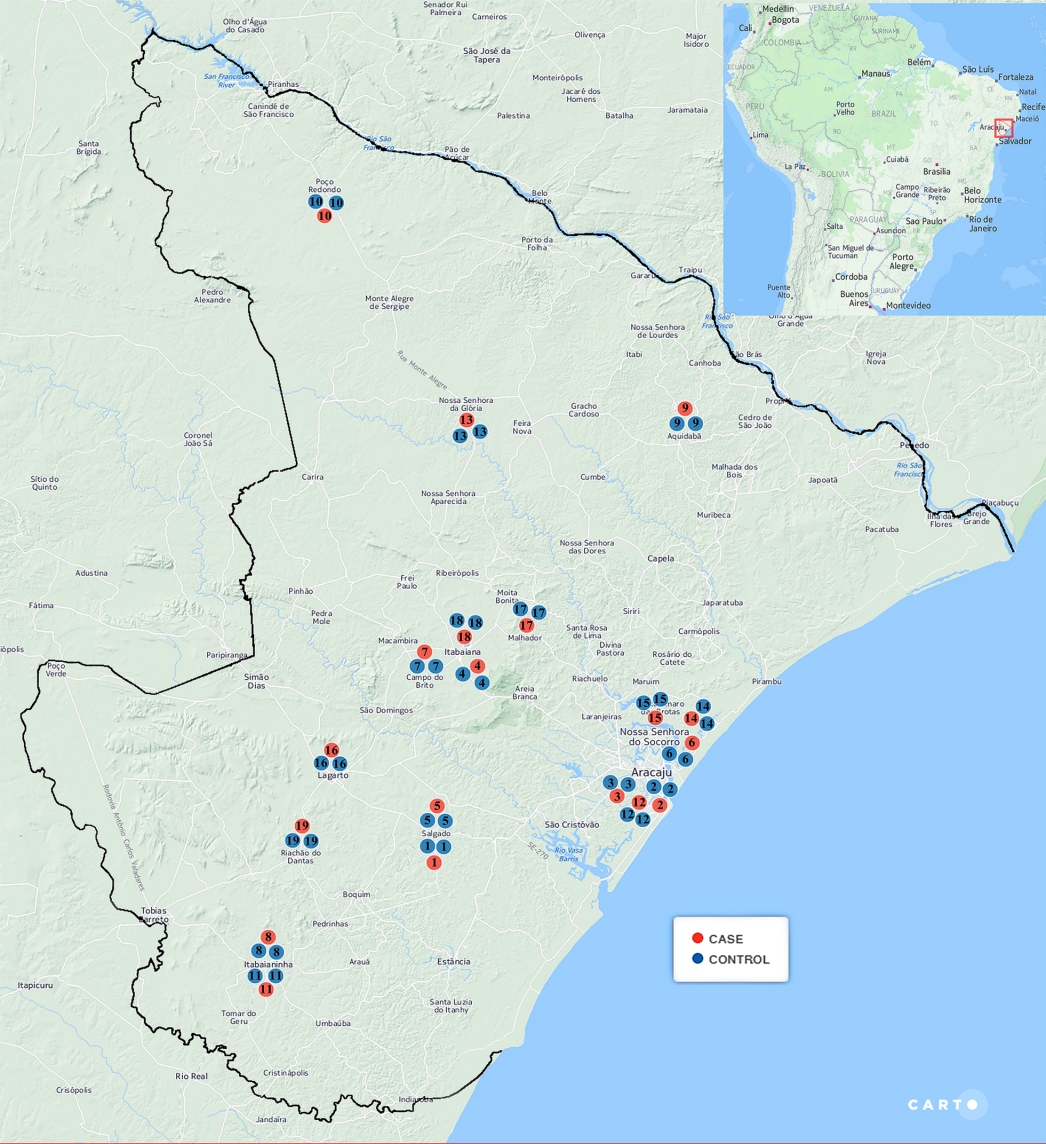
Maps of Sergipe and South America (*insert*) showing the place of residence of each case together with its two matched controls included in the study. Map templete: ©OpenStreetMaps contributors (open-source). ©CartoDB, CartoDB attribution

Each included case was matched to at least two controls by place of residence (city), epidemiological week of the delivery (±4 weeks) and gestational age at birth (±2 weeks). The rationale for the matching was to compare women who had pregnancies in nearby places, during the same period of the year and of similar durations (Fig. 1). The majority of the included cases (30 out of 36) could be paired to controls using the matching criteria (80 controls were selected).

For sample size calculation, we considered that 38% of the individuals exposed to Zika virus (IgM positive) [13] and a much lower proportion of not exposed individuals (2%, theoretical) will experience symptoms. The calculation was performed using the LEE online tool (http://www.lee.dante.br) considering a matched case–control design, two controls per case, α = 0.05 (two sided) and β = 0.8. According to the sample size calculation, 12 cases and 24 controls were required (12 trios of one case and two controls).

From March 15th, 2016 to June 5th, 2016, two pediatricians applied telephone questionnaires to mothers in order to obtain information about the acute signs and symptoms experienced during pregnancy, along with other relevant information (a blank questionnaire can be found in the Additional file 2). The pediatricians were unaware of whether or not the mothers were part of the study’s case or controls groups. Additionally mothers surveyed were not told of the principal reason for the research, simply to avoid any potential bias. Seventy-eight participants were contacted (20 cases and 58 controls), 73 agreed to participate (19 cases and 54 controls) and 19 completed trios of one case and two controls could be formed.

Next, mothers were classified accordingly to the suspected Zika virus case definition criteria of the Pan American Health Organization [14]. The case definition was symptoms of rash combined with two or more of the following signs and symptoms: fever, conjunctivitis (non-purulent/hyperemic), arthralgia, myalgia and periarticular edema.

The variables retrieved from medical records, and included in the questionnaire are presented in Table 1 and their univariate effect over congenital microcephaly was evaluated by conditional logistic regression, or Pair *t* test, when applicable. The association between suspected Zika virus disease and congenital microcephaly was also estimated by conditional logistic regression and expressed as odds ratio with 95% confidence intervals. The statistical significance was defined as a p value of less than 0.05 and the crude odds ratio was adjusted for variables found to be significant at p < 0.15 in the univariate analysis (with exception for those used in the suspected Zika virus classification). Statistical analyses were performed using SAS software (version 9.4) and GraphPad Prism Software (version 6.0).

**Table 1:** Studied variables and exposures for cases and controls

Variable	Cases	Controls	p value
Maternal demographics			
Age—mean (sd)—year	26.95 (7.15)	27.55 (4.71)	0.751
Marital status— not single—no./total no. (%)	9/19 (47.4)	17/38 (44.7)	0.860
Educational level—no./total no. (%)			1
Elementary school	4/19 (21.0)	8/38 (21.0)	
Middle school	6/19 (31.5)	12/38 (31.5)	
High School	9/19 (47.4)	18/38 (47.4)	
Occupation—no./total no. (%)			0.077
Homemaker	16/19 (84.2)	22/38 (57.8)	
Not-homemaker	3/19 (15.7)	16/38 (42.1)	
Past infection by dengue virus—no./total no. (%)	8/19 (42.1)	8/38 (21.0)	0.127
Traveled before symptoms—no./total no. (%)	2/19 (10.5)	1/38 (2.6)	0.258
Risk factors for congenital malformations			
Contact with toxic substances^a^—no./total no. (%)	0/19 (0.0)	5/38 (13.1)	0.337
Smoking—no./total no. (%)	0/19 (0.0)	2/38 (5.2)	0.561
Alcohol consumption—no./total no. (%)	0/19 (0.0)	2/38 (5.2)	0.561
Use of folic acid-based medication—no./total no. (%)	12/19 (63.1)	17/38 (44.7)	0.222
Consanguinity—no./total no. (%)	2/19 (10.5)	4/38 (10.5)	1
Genetic disease in the family—no./total no. (%)	0/19 (0.0)	1/38 (2.6)	0.681
Delivery and neonate data			
Gestational age of birth—mean (sd)—week	37.79 (1.6)	37.89 (1.4)	0.725
Epidemiological week of birth—mean (sd)—week	43.95 (4.8)	43.63 (3.3)	0.656
Type of delivery—vaginal—no./total no. (%)	12/19 (63.1)	21/38 (55.2)	0.505
Head circumference—mean (sd)—cm	28.50 (2.2)	34.40 (0.9)	<0.0001
Thoracic circumference—mean (sd)—cm	30.76 (1.9)	32.84 (1.1)	0.0004
Height—mean (sd)—cm	44.18 (2.3)	47.64 (1.5)	0.0001
Weight—mean (sd)—kg	2.589 (0.42)	3.329 (0.326)	<0.0001
Gender—females—no./total no. (%)	12/19 (63.1)	24/38 (63.1)	1
Symptoms experienced during pregnancy			
Any rash—no./total no. (%)	12/19 (63.1)	7/38 (18.4)	0.005
Macular rash—no./total no. (%)	4/19 (21.0)	5/38 (13.1)	0.429
Maculopapular rash—no./total no. (%)	8/19 (42.1)	2/38 (5.2)	0.009
Fever—no./total no. (%)	10/19 (52.6)	3/38 (7.8)	0.004
Conjunctivitis— no./total no. (%)	4/19 (21.0)	2/38 (5.2)	0.109
Arthralgia—no./total no. (%)	9/19 (47.3)	2/38 (5.2)	0.005
Myalgia—no./total no. (%)	5/19 (26.3)	0/38 (0.0)	0.218
Peri-articular edema—no./total no. (%)	8/19 (42.1)	5/38 (13.1)	0.030
Headache—no./total no. (%)	8/19 (42.1)	6/38 (15.7)	0.039
Retro-orbital pain—no./total no. (%)	4/19 (21.0)	3/38 (7.9)	0.149
Fatigue/malaise—no./total no. (%)	9/19 (47.3)	2/38 (5.2)	0.005
Dizziness—no./total no. (%)	2/18 (11.1)	1/38 (2.6)	0.258
Lymphadenopathy—no./total no. (%)	0/19 (0.0)	1/38 (2.6)	0.681
Mouth sores—no./total no. (%)	0/19 (0.0)	0/38 (0.0)	–
Breathlessness—no./total no. (%)	0/19 (0.0)	1/38 (2.6)	0.681
Diarrhea—no./total no. (%)	1/19 (5.2)	0/38 (0.0)	0.582
Anorexia—no./total no. (%)	6/19 (31.5)	1/38 (2.6)	0.178
Alterations in taste—no./total no. (%)	4/19 (21.0)	1/38 (2.6)	0.275
Cough—no./total no. (%)	2/19 (10.5)	1/38 (2.6)	0.459
Case definition			
Suspected Zika virus disease—no./total no. (%)	10/19 (52.6)	3/38 (7.9)	0.004
Gestational age of symptoms—no./total no. (%)			
1st trimester	5/19 (26.3)	0/38 (0.0)	0.157
2nd trimester	5/19 (26.3)	3/38 (7.9)	
Duration of the symptoms—mean (sd)—days	6.70 (5.08)	9.6 (4.6)	0.258

^a^Toxics substances were paint, insecticide (n = 3) and raticide. p values were calculated by univariate conditional logistic regression or paired t test, when applicable

Results demonstrated that mothers who delivered neonates with congenital microcephaly were more likely to have experienced rash (mainly maculopapular), fever, arthralgia, periarticular edema, headache and fatigue/malaise compared with controls. In addition to the lower head circumference, microcephalic neonates also had lower thoracic circumference, lower heights and lower weights compared to non-microcephalic babies (all in Table 1). No differences were observed for other studied variables and exposures.

Moreover, 10 out 19 (56%) cases versus 3 out of 38 (7.9%) controls met the study’s case definition. Therefore, the odds ratio for suspected Zika virus during pregnancy and subsequently giving birth to a neonate with congenital microcephaly was 9.28 (95% CI 2.02–42.67, p = 0.004). Mother occupation and past infection by dengue virus showed borderline significance in the univariate analysis (p = 0.077 and 0.127, respectively). After the adjustment of these variables, the odds ratio remained significant (OR = 9.85; 95% CI 1.83–53.05, p = 0.008) (Table 2). Additionally, prenatal ultrasound or transfontanellar ultrasound results were available for all 19 cases and 13 (68.4%) presented abnormal findings indicative of Zika virus congenital syndrome (Additional file 1: Table S1) (for review see [15, 16]).

**Table 2:** Estimated risks of microcephaly

Variable	Univariate analysis			Adjusted analysis		
	Odds ratio	95% CI	p value	Odds ratio	95% CI	p value
Suspected Zika virus disease	9.28	2.02–42.67	0.004	9.85	1.83–53.05	0.008
Occupation (homemaker)	4.00	0.86–18.57	0.077	8.33	0.91–76.05	0.06
Past infection by dengue virus	2.45	0.77–7.75	0.127	3.06	0.60–15.57	0.176

p values were calculated by univariate or multivariate (adjusted) conditional logistic regression

In conclusion, mothers who experienced an exanthematous illness displaying Zika-like symptoms during pregnancy had 10 times higher odds of delivering a newborn with congenital microcephaly compared to mothers who did not. Six signs and symptoms experienced by the mothers were associated with congenital microcephaly [Rash (mainly maculopapular), fever, arthralgia, periarticular edema, headache and fatigue/malaise]. Taken together, they are similar to the suspected Zika-virus case definition considered for the study. These findings suggest that the risk of congenital microcephaly could be predicted by analyzing these clinical signs and symptoms. Because Zika virus diagnosis is not readily available, as is often the case in poor underdeveloped regions, or can be a significant challenge: (a) the RT-qPCR assay has a limited detection window because the virus is present for 11–17 days in the blood [17] and negative results may not exclude the infection [18]; (b) the available immunologic tests are not definitive since false-positives and cross-reaction due past flaviviruses infection or vaccination might occur [18]. Thus, health care providers could estimate the risk of delivering a newborn with microcephaly assessing the clinical signs and symptoms that the pregnant woman experienced during the gestation or evaluating if the pregnant woman meets the suspected Zika virus definition laid out in this study. Moreover, microcephalic overall anthropometries were lower than non-microcephalic suggesting intrauterine growth restriction (as observed in animal models infected with Zika virus [19]). Homemakers, as an occupation, and past dengue virus history seem to also play a role in the risk of microcephaly. Indeed, today *Aedes Aegypti* mosquitoes have adapted to deposit their eggs in domestic water and to feed on humans [7], so being a homemaker could potentially, by nature of the work, increase the exposure to arboviruses, and Zika virus infection could be enhanced by dengue antibodies [20].

On the other hand, as the present study was designed to locate the association between the suspected Zika virus during pregnancy and the delivery of a newborn with congenital microcephaly, further research with more statistical power is needed to draw definitive conclusions for the other signs, symptoms and exposures described in Table 1 that almost reached statistical significance (e.g. conjunctivitis and retro-orbital pain).

Because Zika, dengue and chikungunya viruses cause diseases with similar clinical symptoms, and they have circulated in northeastern Brazil in 2015, we could not distinguish precisely which one caused the acute exanthematous illness in the study subjects. However, evidence suggests that Zika virus was the likeliest etiology for the exanthematous illness outbreak. A report including 77 samples from patients with acute exanthematous illness collected in Tuparetama, Pernambuco, during the 2015 outbreak revealed that Zika virus was present in 40.2%, dengue virus in 11.7% and chikungunya virus 1.2% and coinfection of Zika and dengue viruses was also reported in 2.6% [1]. Similarly, a second study including 24 samples from Camaçari, Bahia, found that 29.2, 0 and 12.5% tested positive for Zika, dengue and chikungunya viruses, respectively [3]. Additionally, abnormal ultrasound findings compatible with the Zika virus congenital syndrome were found in most of cases. Taken together, these evidences support the assumption that majority of the included cases were exposed to Zika virus.

In conclusion, the objective of the study was reached; the acute exanthematous illness outbreak was associated with the congenital microcephaly outbreak. This knowledge could have helped to limit some of the misquided speculation and could have expedited public health policies more effectively targeting the mosquito vector. A deeper understanding of the specific microcephaly cause would be a next step.

## Limitations

The limitations of the present study were: (a) participants recall bias, (b) absence of laboratory test results for Zika virus and other arboviruses and (c) incomplete test results for other pathogens that could lead to microcephaly. Regarding the participants recall bias, the use of “blind” surveys is considered the most effective device to reduce potential bias. Neither the mothers or the interviewers knew who was a member of the case or control groups [21]. The absence of laboratory testing results for Zika virus made it impossible to confirm the viral infection in the volunteers and its causal relation with microcephaly. The absence of laboratory test results for other arboviruses also made it challenging to correctly define the specific etiological agent of the exanthematous illness experienced by some mothers. Finally, the incomplete test results for other pathogens that could lead to microcephaly such as rubella, toxoplasmosis and cytomegalovirus indicate that these agents cannot be excluded as causes of congenital microcephaly in the studied newborns. Congenital rubella has been eradicated in Brazil [22] and microcephaly is uncommon in congenital toxoplasmosis [15]. Indeed, all these traditional microcephaly etiological agents could not explain the substantial increase of the condition observed in Brazil’s northeastern region in late 2015.

## Additional files

**Additional file 1: Table S1.** Pregnancy data regarding signs and simptoms suggestive of zika virus infection for the 19 cases (or their neonates) included in the study.

**Additional file 2.** Questionnaire.
