# Supplementary material for: Association between suspected Zika virus disease during pregnancy and giving birth to a newborn with congenital microcephaly: a matched case–control study
Source: BMC Res Notes. 2017 Sep 6;10:457. doi: 10.1186/s13104-017-2796-1 (PMC5588708; doi:10.1186/s13104-017-2796-1)
Supplement: Supplementary file 2 — Additional file 2. Questionnaire. [file 13104_2017_2796_MOESM2_ESM.docx]

**Questionnaire**

Instructions

* Identify yourself as pediatrician.

* Explain that you are conducting a research about signs and symptoms developed during pregnancy.

* Confirm if the mother agree in participate from the research by answering some questions. Yes ( ) No ( )

* Verify if the mother delivered a newborn at “Nossa Senhora de Lourdes” maternity hospital.

* Check the name of the volunteer and other information provided by the principal investigation.

Information provided by the principal investigator

Mother

Name:

Date of birth:

Telephone 1:

Telephone 2:

Address:

Neighborhood:

City:

State:

ZIP:

Occupation:

Marital status:

Newborn

Gender:

Date of birth:

Ask the following questions to the mother explaining each sign or symptom using simple words to obtain an effective communication.

During the pregnancy do you experienced rash? Yes ( ) No ( )

Macular Yes ( ) No ( )

Maculopapular Yes ( ) No ( )

Other:_____________________

The rash was accompanied of pruritus? Yes ( ) No ( )

Did you experience the following signs and symptoms at the same period?

Fever Yes ( ) No ( )

Conjunctivitis Yes ( ) No ( )

Arthralgia Yes ( ) No ( )

Myalgia Yes ( ) No ( )

Peri-articular edema Yes ( ) No ( )

Headache Yes ( ) No ( )

Retro-orbital pain Yes ( ) No ( )

Fatigue/malaise Yes ( ) No ( )

Dizziness Yes ( ) No ( )

Lymphadenopathy Yes ( ) No ( )

Mouth sores Yes ( ) No ( )

Breathlessness Yes ( ) No ( )

Diarrhea Yes ( ) No ( )

Anorexia Yes ( ) No ( )

Alterations in taste Yes ( ) No ( )

Cough Yes ( ) No ( )

How long did the signs and symptoms last? ___________Days

Did you travel before the sign and symptoms onset? Yes ( ) No ( )

City/Estate:___________________________________________________________________

Have you ever had dengue fever (before pregnancy, not during)? Yes ( ) No ( )

During the pregnancy did you use alcohol or drugs? Yes ( ) No ( )

During the pregnancy did you take folic acid-based medication? Yes ( ) No ( )

During the pregnancy did you enter in contact with toxic substances (give some examples - solvents, pesticides)? Yes ( ) No ( )

Does your family have history of some genetics disease associated with congenital malformations? Yes ( ) No ( )

Researcher responsible for the interview

Name: _______________________________________________________________________

Date _______/______/_______
